# Supplementary material for: Hybrid Models and Biological Model Reduction with PyDSTool
Source: PLoS Comput Biol. 2012 Aug 9;8(8):e1002628. doi: 10.1371/journal.pcbi.1002628 (PMC3415397; doi:10.1371/journal.pcbi.1002628)
Supplement: Text S4 — Complete source code for the PyDSTool package (version 0.88.120504). Includes API documentation and help files linking to web pages. This file is identical to the current public release on Sourceforge.net. (ZIP) [file pcbi.1002628.s004.zip › PyDSTool/html/PyDSTool.Toolbox.FR-pysrc.html]

xml version="1.0" encoding="ascii"?


PyDSTool.Toolbox.FR


| Home | Trees | Indices | Help | PyDSTool |
| --- | --- | --- | --- | --- |

Package PyDSTool ::
Package Toolbox ::
Module FR

[hide private]

[frames] | no frames]

# Source Code for Module PyDSTool.Toolbox.FR

```
  1  # Fruchterman-Reingold graph drawing algorithm in Python
 
  2  # R. Clewley, November 2005
 
  3  
 
  4  from __future__ import division 
  5  from numpy import array, alltrue, arange, sign 
  6  from numpy.linalg import norm 
  7  import math 
  8  from numpy.random import uniform, normal 
  9  from PyDSTool import interp1d, sortedDictLists 
 10  
 


11 -class vertex(object):


12 -    def __init__(self, x, y):


13          self.pos = array([x, y], 'd') 
 14          self.disp = array([0., 0.])

 15  
 


16 -    def __eq__(self, other):


17          return alltrue(self.pos == other.pos)

 18  
 


19 -    def __neq__(self, other):


20          return not self.__eq__(other)

 21  
 
 22  
 


23 -class edge(object):


24      """v -> u directed edge""" 


25 -    def __init__(self, u, v):


26          self.u = u #vertex(u.pos[0], u.pos[1]) 
 27          self.v = v #vertex(v.pos[0], v.pos[1])

 28  
 


29 -    def __eq__(self, other):


30          return self.u == other.u and self.v == other.v

 31  
 


32 -    def __neq__(self, other):


33          return not self.__eq__(other)

 34  
 
 35  
 
 36  
 
 37  # very simple cooling scheme
 


38 -def cool_simple(t, t0, cfac):


39      return t*math.exp(-cfac*(t0-t)-0.05)

 40  
 
 41  
 


42 -class cool(object):


43 -    def __init__(self, t0, tarray):


44          n = len(tarray)-1 
 45          trange = arange(0., 1+1./n, 1./n) 
 46          self.coolfn = interp1d(trange, t0*tarray) 
 47          self.t0 = t0

 48  
 


49 -    def __call__(self, t):


50          # temp t = t0 -> 0, rescaled to 0 -> 1
 
 51          return self.coolfn((self.t0-t)/self.t0)

 52  
 
 53  
 
 54  tarray = array([0.98, 0.92, 0.86, 0.78, 0.72,
 
 55                  0.67, 0.64, 0.60, 0.50, 0.45,
 
 56                  0.40, 0.35, 0.30, 0.26, 0.20,
 
 57                  0.16, 0.12, 0.09, 0.04, 0.00]) 
 58  
 
 59  # for testing purposes
 


60 -def itercool(t, t0, n=30):


61      cooler = cool(t0, tarray) 
 62      for i in range(n+1): 
 63          print t 
 64          t=cooler(t)

 65  
 
 66  
 


67 -def out_degree(V, E):


68      outd = {} 
 69      for vn in V: 
 70          try: 
 71              outd[vn] = len(E[vn]) 
 72          except KeyError: 
 73              outd[vn] = 0 
 74      return outd

 75  
 


76 -def in_degree(V, E):


77      ind = {} 
 78      for vn, vv in V.iteritems(): 
 79          count = 0 
 80          for vert in V.keys(): 
 81              try: 
 82                  es = E[vert] 
 83              except KeyError: 
 84                  continue 
 85              for e in es: 
 86                  if vv == e.u: 
 87                      count += 1 
 88          ind[vn] = count 
 89      return ind

 90  
 
 91  
 


92 -def FR(V, E, W=[0, 1], L=[0, 1], num_its=30, fixed=[]):


93      """Fruchterman - Reingold graphing algorithm.
 
 94  
 
 95      Changes V and E in-place.""" 
 96  
 
 97      area = (W[1]-W[0]) * (L[1]-L[0]) 
 98      k = math.sqrt(area/len(V)) 
 99  
 
100      def fa(x): 
101          return x*x/k

102  
 
103      def fr(x): 
104          return k*k/x 
105  
 
106      od = out_degree(V, E) 
107      fixedverts = fixed.values() 
108  
 
109      # initial temperature
 
110      t = t0 = max([W[1]-W[0], L[1]-L[0]])/3.5 
111  #    coolfac = 1./num_its
 
112      # num_its unused now -- assumed 30
 
113      cooler = cool(t0, tarray) 
114  
 
115      for i in range(num_its): 
116  #        print "t = %.4f"%t
 
117          # calculate repulsive forces (with weak Gaussian noise)
 
118          for vname, v in V.iteritems(): 
119              # each vertex has two vectors: .pos and .disp
 
120              v.disp = array([0., 0.]) 
121              if vname in fixed: 
122                  continue 
123              for uname, u in V.iteritems(): 
124                  if u != v: 
125                      D = v.pos - u.pos 
126                      aD = norm(D) 
127                      try: 
128                          v.disp = v.disp + (D/aD) * fr(aD) \
 
129                              + array([normal(0, t), normal(0, t)]) 
130                      except ZeroDivisionError: 
131  #                        print "Clipping to graphics frame caused vertices to",\
 
132  #                              "coalesce on iteration %d"%i
 
133  #                        print " @ v.pos=(%.4f,%.4f), u.pos=(%.4f,%.4f)"%(v.pos[0],
 
134  #                                v.pos[1],u.pos[0],u.pos[1])
 
135  #                        print "Trying again..."
 
136                          dx = uniform(W[0],W[1]) 
137                          dy = uniform(L[0],L[1]) 
138                          du = array([dx/3., dy/3.]) 
139                          u.pos = u.pos + du 
140                          D = v.pos - u.pos 
141                          aD = norm(D) 
142                          v.disp = v.disp + (D/aD) * fr(aD) \
 
143                               + array([normal(0, t), normal(0, t)]) 
144          for vname, v in V.iteritems(): 
145              # penalize edges from common vertex that are very close to
 
146              # eachother in angle
 
147              if od[vname] > 2: 
148                  angle_dict = {} 
149                  for e in E[vname]: 
150                      D = e.u.pos - e.v.pos 
151                      try: 
152                          angle_dict[e] = math.atan(D[1]/D[0]) 
153                      except ZeroDivisionError: 
154                          sgnD = sign(D[1])  # get weird errors from this sometimes 
155                          if sgnD > 0: 
156                              angle_dict[e] = math.pi/2 
157                          else: 
158                              angle_dict[e] = -math.pi/2 
159                  angles_sorted, vals_sorted = sortedDictLists(angle_dict) 
160                  num_outs = od[vname] 
161                  for i in range(num_outs): 
162                      dtheta1 = angle_dict[angles_sorted[i]] - angle_dict[angles_sorted[i-1]] 
163                      ed = angles_sorted[i].u.pos - angles_sorted[i].v.pos 
164                      if abs(dtheta1) < 0.4: 
165                          # swap x, y of target vertex to get orthogonal direction
 
166                          # (rotating in -ve angle direction)
 
167                          vd = array([ed[1], -ed[0]]) 
168                          vd = vd/norm(vd) 
169                          try: 
170                              c = max([0.07, 0.03/abs(dtheta1)]) 
171                          except ZeroDivisionError: 
172                              c = 0.07 
173                          angles_sorted[i].u.disp = angles_sorted[i].u.disp - c*vd 
174                          # angles are close enough that use same direction for other vertex
 
175                          angles_sorted[i-1].u.disp = angles_sorted[i-1].u.disp + c*vd 
176                      ni = (i+1)%num_outs 
177                      dtheta2 = angle_dict[angles_sorted[ni]] - angle_dict[angles_sorted[i]] 
178                      if abs(dtheta2) < 0.4: 
179                          # swap x, y of target vertex to get orthogonal direction
 
180                          # (rotating in -ve angle direction)
 
181                          vd = array([ed[1], -ed[0]]) 
182                          vd = vd/norm(vd) 
183                          try: 
184                              c = max([0.07, 0.03/abs(dtheta2)]) 
185                          except ZeroDivisionError: 
186                              c = 0.07 
187                          angles_sorted[i].u.disp = angles_sorted[i].u.disp + c*vd 
188                          # angles are close enough that use same direction for other vertex
 
189                          angles_sorted[ni].u.disp = angles_sorted[ni].u.disp - c*vd 
190          # calculate attractive forces along edges
 
191          for vname, elist in E.iteritems(): 
192              if od[vname] > 3: 
193                  att_scale = 1.75 
194              else: 
195                  att_scale = 2.5 
196              for e in elist: 
197                  # each edge is an ordered pair of vertices .v and .u
 
198                  D  = e.v.pos - e.u.pos 
199                  aD = norm(D) 
200                  disp = (D/aD) * att_scale*fa(aD) 
201                  if e.v not in fixedverts: 
202                      e.v.disp = e.v.disp - disp 
203                  if e.u not in fixedverts: 
204                      e.u.disp = e.u.disp + disp 
205  
 
206          # limit the maximum displacement to the temperature t
 
207          # and then prevent vertex being displaced outside frame
 
208          for v in V.itervalues(): 
209              if v in fixedverts: 
210                  continue 
211              v.pos = v.pos + (v.disp/norm(v.disp)) * min([norm(v.disp), t]) 
212              v.pos[0] = min([W[1], max([W[0], v.pos[0]])]) 
213              v.pos[1] = min([L[1], max([W[0], v.pos[1]])]) 
214          # reduce the temperature as the layout approaches a better configuration
 
215          t = cooler(t) 
216
```

  


| Home | Trees | Indices | Help | PyDSTool |
| --- | --- | --- | --- | --- |

|  |  |
| --- | --- |
| Generated by Epydoc 3.0.1 on Fri May 4 15:24:12 2012 | http://epydoc.sourceforge.net |
